# Fluorescent Nanocomposite Hydrogels Based on Conjugated Polymer Nanoparticles as Platforms for Alkaline Phosphatase Detection

**DOI:** 10.3390/bios13030408

**Published:** 2023-03-21

**Authors:** Yolanda Alacid, Rocío Esquembre, Francisco Montilla, María José Martínez-Tomé, C. Reyes Mateo

**Affiliations:** 1 Instituto de Investigación, Desarrollo e Innovación en Biotecnología Sanitaria de Elche (IDiBE), Universidad Miguel Hernández, 03202 Elche, Spain; 2 Departamento de Química Física and Instituto Universitario de Materiales de Alicante (IUMA), Universidad de Alicante, 03690 Alicante, Spain

**Keywords:** nanocomposite hydrogel, polyfluorene, fluorescent sensor, alkaline phosphatase, immobilization, portable device, enzyme

## Abstract

This work describes the development and characterization of fluorescent nanocomposite hydrogels, with high swelling and absorption capacity, and prepared using a green protocol. These fluorescent materials are obtained by incorporating, for the first time, polyfluorenes-based nanoparticles with different emission bands—poly[9,9-dioctylfluorenyl-2,7-diyl] (PFO) and poly[(9,9-di-n-octylfluorenyl-2,7-diyl)-alt-(1,4-benzo-{2,1,3}-thiadiazole)] (F8BT)—into a three-dimensional polymeric network based on polyacrylamide. To this end, two strategies were explored: incorporation of the nanoparticles during the polymerization process (in situ) and embedment after the hydrogel formation (ex situ). The results show that the combination of PFO nanoparticles introduced by the ex situ method provided materials with good storage stability, homogeneity and reproducibility properties, allowing their preservation in the form of xerogel. The fluorescent nanocomposite hydrogels have been tested as a transportable and user-friendly sensing platform. In particular, the ability of these materials to specifically detect the enzyme alkaline phosphatase (ALP) has been evaluated as a proof-of-concept. The sensor was able to quantify the presence of the enzyme in an aqueous sample with a response time of 10 min and LOD of 21 nM. Given these results, we consider that this device shows great potential for quantifying physiological ALP levels as well as enzyme activity in environmental samples.

## 1. Introduction

Polymeric hydrogels are materials composed of an aqueous phase and one or more solid continuous phases. The continuous phase generates a cross-linked network, inside which are the water molecules, through covalent or non-covalent bonds that provide structural stability and prevent the dissolution of the polymer in the aqueous medium. The result is a viscoelastic material, similar to a solid, but highly deformable [1]. Among the family of hydrogels, those based on acrylamide and related polymers form lightly cross-linked tridimensional networks which swell strongly in water, showing good biocompatibility and optical transparency [2,3].

Hydrogel properties can be easily modulated and/or improved. This is the case of nanocomposite hydrogels (NCHs), which are manufactured by incorporating nanomaterials inside polymeric materials, such as nanoparticles of silica, gold or silver, carbon nanotubes, graphene, magnetic nanoparticles or quantum dots [4,5]. This innovative strategy results in a synergistic effect that improves the properties of both components and generates multifunctional hydrogels with varied characteristics, such as greater elasticity and resistance (compared to traditionally manufactured hydrogels), higher electrical and thermal conductivity, magnetic properties, color change or luminescence [6,7].

The incorporation of fluorescent nanoparticles in hydrogels gives these materials luminescent properties that can be exploited, besides other applications, for the development of optical sensing devices [8,9]. Among the fluorescent nanomaterials incorporated in hydrogels, the most commonly used are semiconductor quantum dots (QDs); this is due to their high quantum yield, and their ease of modulating optical properties based on size and composition [10]. Recent works have developed fluorescent sensors incorporating QDs in hydrogels for the detection of glucose and progesterone [11,12]; however, these fluorescent NCHs present the limitation of the QDs toxicity due to the heavy elements they contain. Carbon-based nanomaterials, such as carbon quantum dots (CDs) and graphene quantum dots (GQDs), have grown quickly in recent years as new fluorescent nanomaterials, which show much larger biocompatibility than QDs and lower toxicity; however, they also show lower fluorescence efficiency [13,14]. Embedded in hydrogels, they have been mainly used for rapid heavy metal ion and polyaromatic compound detection [15,16]. The nano-structuring of hydrogels with fluorescent liposomes has also been described, though these materials have been intended for drug delivery applications rather than sensor development [17].

Recently, conjugated polymer-based nanoparticles (CNPs) have emerged as a new class of fluorescent nanoparticle with interesting properties, such as good biocompatibility, excellent photostability and high quantum efficiency; CNPs also offer the possibility of tunable fluorescence emission by appropriate design of the polymer molecular backbone [18,19,20,21]. In addition, conjugated polymers, especially those involving fluorene-based systems (known as polyfluorenes), are excellent electron and energy donors; this means that the fluorescence of CNPs can be quenched with suitable acceptors, which gives them great versatility as sensing elements [22,23]. In this regard, micellar nanoparticles based on novel polyfluorene polyelectrolytes have been developed in recent work, as promising materials for the detection of pollutants through photoinduced electron transfer and inner filter effect mechanisms [24]. In that work, fluorescent hydrogels were also developed by incorporating the synthesized polyelectrolytes, but not the micellar nanoparticles, into the hydrogels. Conjugated polymers have also been successfully used as precursors to build fluorescent supramolecular cross-linked polymer networks for sensing purposes [25]. However, fluorescent materials obtained by incorporating CNPs into hydrogels, to obtain fluorescent NCHs, have hardly been reported. Recently, CNPs emitting red, green and blue fluorescence were covalently embedded in poly(N-isopropylacrylamide) (PNIPAM) hydrogel to obtain a thermosensitive nanocomposite [26]. Our group also successfully incorporated multicolor fluorescent conjugated polyelectrolytes in pH-sensitive nanogels of poly (methyl vinyl ether-alt-maleic acid) monoethyl ester (PMVEMA), which were applied for drug transport and bioimaging [27]. However, to our knowledge, no studies have been published in which these fluorescent nanocomposites have been used as sensing platforms for trace detection and quantification of compounds of clinical and environmental interest.

**Scheme 1:**
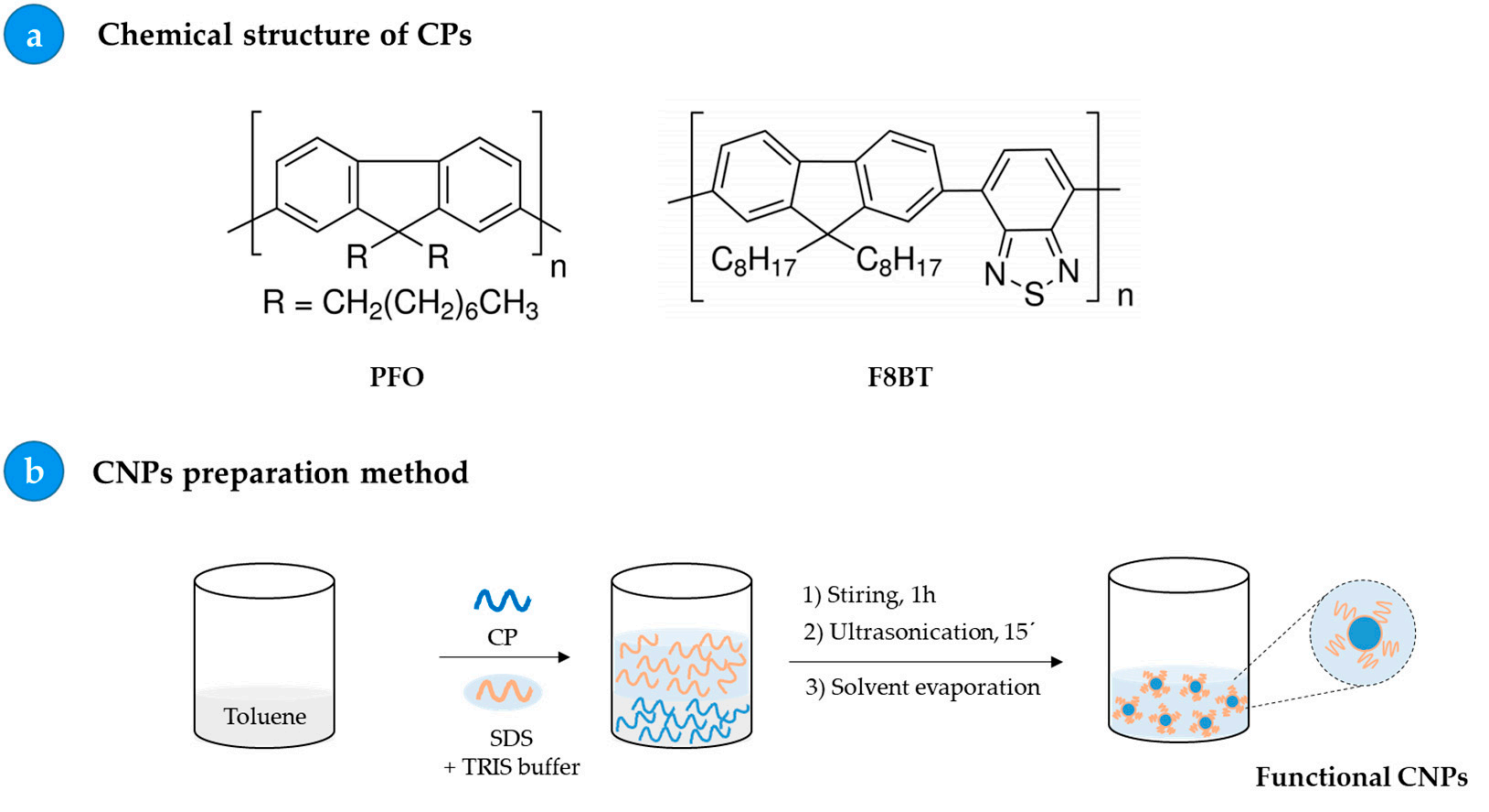
(**a**) Chemical structure of the conjugated polymers (CPs): PFO and F8BT. (**b**) Preparation procedure of fluorescent nanoparticles from PFO and F8BT.

This work describes the design and characterization of fluorescent NCHs prepared through a green route, which can be swollen and dried without degradation. These fluorescent materials are obtained by embedding polyfluorenes-based CNPs into the polymer matrix, which endows the hydrogels with favorable fluorescence and potential applications in sensing and biosensing. In particular, we have taken advantage of the optical transparency and swelling properties of acrylamide hydrogels to incorporate CNPs made from two polyfluorene derivatives with different emission bands: poly[9,9-dioctylfluorenyl-2,7-diyl] (PFO), and poly[(9,9-di-n-octylfluorenyl-2,7-diyl)-alt-(1,4-benzo-{2,1,3}-thiadiazole)] (F8BT), an alternating copolymer of fluorene and benzothiadiazole (Scheme 1). Hydrogels were synthesized via a simple and rapid protocol through UV light-induced radical polymerization of the cationic monomer [2-(acryloyloxy)ethyl]trimethylammonium chloride (AETA), and N,N′-methylenebis(acrylamide) (MBA) as a cross-linker. This type of hydrogel, described by Martin Pacheco et al. [16], is cationic in nature and was recently used by our group to immobilize enzymes for the development of colorimetric and amperometric biosensors [28,29].

As a sensing application, the ability of the hydrogel incorporating PFO nanoparticles (PFO@AETA) to detect alkaline phosphatase (ALP) was explored. This enzyme is associated with many human diseases, making its quantitative detection vital from a clinical perspective [30]. In addition, accurate detection of the amount of ALP in water is also of great importance for ecological environments and human production activities, and its concentration provides an indicator of water quality [31]. Recent work has described the detection of ALP in aqueous media through a ratiometric sensing system, using fluorescent nanohybrids based on polyfluorenes [32]. In our work, the sensing mechanism is based on the potential quenching of PFO@AETA by *p*-nitrophenol (PNP), the end product of hydrolysis of *p*-nitrophenyl phosphate (PNPP) catalyzed by ALP. PNP is an electron acceptor that absorbs at 405 nm in its anionic form, and has been described by our laboratory to quench the fluorescence intensity of cationic polyfluorenes. This process is presumed to take place via a combination of photoinduced electron transfer, fluorescence resonance energy transfer and inner filter mechanisms [22,33].

## 2. Materials and Methods

### 2.1. Materials and Reagents

Hydrogels were synthesized by radical polymerization of [2-(acryloyloxy)ethyl]trimethylammonium chloride (AETA) in the presence of *N*,*N*’-methylenebis(acrylamide) (MBA), which acted as the crosslinking agent, and 2,4,6-lithium trimethylbenzoylphenylphosphinate (LiTPO), which acted as the photoinitiator; all chemicals were purchased from Sigma-Aldrich (Merck Life Science, Madrid, Spain). The conjugated polymers poly[9,9-dioctylfluorenyl-2,7-diyl] (PFO) and poly[(9,9-dioctylfluorenyl-2,7-diyl)-alt-co-(1,4-benzo-{2,1,3}-thiadiazole)] (F8BT) were obtained from American Dyes Source, Inc. The enzyme alkaline phosphatase (ALP) (EC 3.1.3.1; from bovine intestinal mucosa; lyophilized powder; M_w_ = 140,000 Da), the substrate *p*-nitrophenyl phosphate (PNPP), the fluorescence quencher *p*-nitrophenol (PNP) and sodium dodecyl sulfate (SDS) were purchased from Sigma-Aldrich (Merck Life Science, Madrid, Spain). Stock solutions of ALP, PNPP and PNP were prepared in TRIS buffer (55 mM, pH 9) and stored at 4 °C at 45.1 µM, 2 mM and 1 mM, respectively. TRIS buffer (55 mM, pH 9) was prepared by mixing TRIS base and hydrochloric acid with Milli-Q water using Milli-Q equipment (Millipore, Madrid, Spain). All other solvents were of spectroscopic or analytical reagent grade (Uvasol; Merck, Madrid, Spain).

### 2.2. Obtention of AETA Hydrogels

The protocol previously described by Alacid et al. [28] was followed to synthesize AETA hydrogels. In this experiment, 5.66 mL of AETA (26.4 mmol) and 0.01 g of MBA (0.06 mmol) were combined with 5 mL of Milli-Q water, and then LiTPO (0.02 g, 0.06 mmol) was added to the mixture. The solution was homogenized by stirring under dark conditions, and then drawn through a disposable syringe and exposed to UV light (𝜆 = 365 nm) for 1 min. The resulting hydrogel was removed from the container and further treated by immersion in distilled water for 5 days to remove any residual monomer or initiator. Finally, the hydrogel was dried by heating it in an oven at 45 °C for 2 days.

### 2.3. Preparation of Fluorescent Nanoparticles: PFO_CNPs and F8BT_CNPs

Fluorescent nanoparticles were prepared in aqueous medium from the conjugated polymers PFO and F8BT, respectively, following the protocol already described by Pecher et al. [34] and summarized in Scheme 1b. For this purpose, 5 mL of a 10 mg/mL solution of polymer in toluene was mixed with 10 mL of a 55 mM TRIS buffer solution and 4 mg/mL SDS. It was shaken for 1 h with a magnetic stirrer in a closed vial. The sample was placed in an ultrasound probe, power 125W, using a QSonica model Q125 sonicator (QSonica, Newtown, CT, USA) for 15 min, with 90% amplitude; at this stage, we kept the vial with the solution submerged in ice water to avoid heating and foam production. It was then heated to 60 °C to evaporate the organic solvent. Finally, the solution was stored at 4 °C.

### 2.4. Obtention of PFO@AETA and F8BT@AETA Hydrogels

**Scheme 2:**
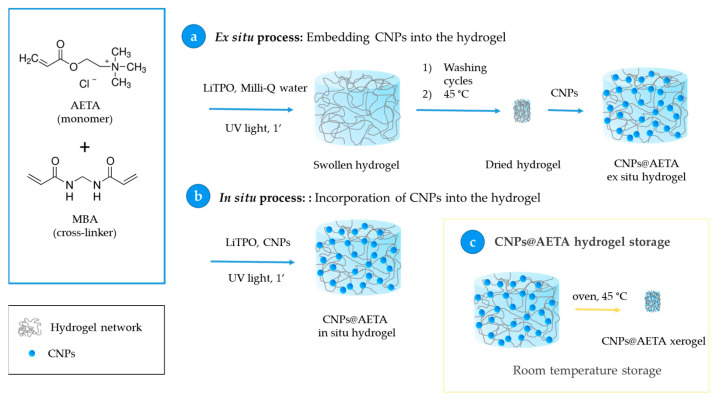
Fabrication of fluorescent hydrogels based on the incorporation of CNPs ex situ (**a**) and in situ (**b**). (**c**) Fabrication of CNPs@AETA xerogels for hydrogel storage.

To obtain fluorescent nanocomposite hydrogels, the developed CNPs were incorporated in AETA hydrogels using two adaptations of the same procedure, as shown in Scheme 2 and described in this list:

- Ex situ process: Embedment of the CNPs into the hydrogel. In this process, 0.07 g of hydrogel prepared by the above method was immersed in 1.8 mL of a 6 µM solution of already prepared CNPs for 24 h, to ensure that all the solution was completely absorbed, and then stored at 4 °C. This step allows the total loading of the fluorescent nanoparticles from the CNPs solution into the hydrogel network (Scheme 2a).
- In situ process: Incorporation of CNPs into the hydrogel. Nanoparticles of PFO and F8BT were incorporated before the polymerization process by adding 5 mL of a solution of CNPs 6 µM to the AETA-MBA-LiTPO mixture. After the irradiation with UV light, fluorescent nanocomposite hydrogels were obtained (Scheme 2b).

To ensure convenient storage of the hydrogels, a process of dehydration was carried out by heating in an oven (45 °C, 48 h). This resulted in the transformation of the hydrogels into xerogels (Scheme 2c).

### 2.5. Absorbance Measurements

Absorbance measurements of CNPs were carried out at room temperature using a model UV-2700 spectrophotometer (Shimadzu, Tokyo, Japan). Quartz cuvettes of 10 × 10 mm were used. Absorbance spectra were collected between wavelengths of 250 and 600 nm.

### 2.6. Steady-State Fluorescence Measurements

Steady-state fluorescence measurements, both in solution and in hydrogels, were performed in 10 × 10 mm quartz cuvettes using a PTI-QuantaMaster spectrofluorometer (PTI, Birmingham, NJ, USA) interfaced with a Peltier cell. Fluorescence emission spectra were collected by exciting the samples at 396 or 466 nm for PFO and F8BT CNPs, respectively. The fluorescence intensity of the blanks (hydrogel without CNPs) was checked and subtracted from the samples. For the thermal stability study, heating rates of 5 °C min^−1,^ and stabilization times of 30 s at each temperature, were selected.

### 2.7. Time-Resolved Fluorescence Measurements

The time-resolved fluorescence measurements of PFO and F8BT were carried out by a high-performance fluorescence lifetime spectrometer FluoTime 300 (PicoQuant, Berlin, Germany), with time-correlated single-photon counting (TCSPC). For PFO CNPs and PFO@AETA, a 372 nm picosecond pulsed diode laser (LDH-P-C-375) was used as the excitation source, while the fluorescence decays were collected at 465 nm. For F8BT CNPs and F8BT@AETA samples, a 450 nm picosecond pulsed diode laser (LDH-P-C450) and a detection wavelength of 533 nm were used. Data analysis was performed using the EasyTau software (PicoQuant, Berlin, Germany) and applying a tri-exponential decay model. From the fitting of recovered parameters, the intensity-weighted mean lifetimes were calculated according to:

<τ> = (∑a_i_·τ_i_^2^)/(∑a_i_·τ_i_) (1)

where a_i_ and τ_i_ are the amplitudes and lifetimes, respectively, of the individual components.

### 2.8. Swelling Measurements

Swelling measurements of the xerogels were carried out in the absence or presence of PFO or F8BT CNPs, utilizing either ex situ or in situ methodology. The dried hydrogels, weighing approximately 0.07 g and measuring 0.3 cm in diameter and 0.6 cm in size, were placed in an excess of Milli-Q water and stored at room temperature. The initial weight of each sample was recorded, and subsequent measurements were taken periodically as the hydrogels swelled and until a stable weight was reached. The swelling degree (SW) was calculated using the following formula:

(2) $$\mathrm{SW}=\;\frac{W_{t}-W_{0}}{W_{0}}$$

where *W_t_* represents the weight of the hydrogel at time *t*, and *W*_0_ represents the initial weight of the dry hydrogel.

### 2.9. Particle Size and Zeta Potential

The size and zeta potential of PFO and F8BT CNPs were analyzed by the dynamic light scattering (DLS) technique, using a Malvern Zetasizer Nano-ZS instrument (Worcestershire, UK) equipped with a monochromatic coherent 4 mW helium neon laser (λ = 633 nm) light source; size measurements were performed at angles of 173°. Size was measured in disposable cuvettes, while zeta potential measurements were performed in specific zeta potential cells. All measurements were carried out in triplicate at room temperature.

### 2.10. Scanning Electron Microscope

Scanning electron microscopy (SEM) was performed by using the Sigma 300 VP model (Carl Zeiss Microscopy GmbH, Oberkochen, Germany) Schottky-type field emission-scanning electron microscope (FESEM) at low kV without coating. Images of PFO and F8BT CNPs were obtained by placing a drop of the corresponding suspension onto a silicon wafer. These samples were air-dried before being placed in the microscope.

## 3. Results and Discussion

### 3.1. Preparation and Characterization of PFO_CNPs and F8BT_CNPs

**Figure 1:**
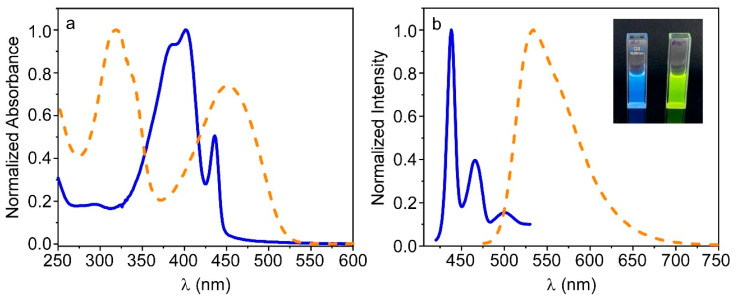
Normalized UV-Vis absorption (**a**) and fluorescent emission spectra (**b**) of PFO_CNPs (blue solid line) (𝜆_x_ = 396 nm) and F8BT_CNPs (orange dashed line) (𝜆_x_ = 466 nm) in buffer. Inset in (**b**) shows a picture of the PFO (left) and F8BT (right) fluorescent CNPs.

PFO and F8BT (Scheme 1a) are well-known neutral polyfluorenes that are completely immiscible in water, which greatly limits their potential biological and environmental applications. The use of nanoparticles based on these compounds, such as those prepared in this work (Scheme 1b), has made it possible to overcome this limitation, allowing for their extensive characterization in aqueous media. These polymers were selected because they emit in different spectral regions, allowing this study to obtain fluorescent hydrogels with different emission bands. Figure 1 shows the absorption and fluorescence emission spectra of both PFO and F8BT CNPs in TRIS buffer, while Figure S1 also displays the emission spectra of the polymers in chloroform. As expected, both nanoparticles emit at different wavelengths, showing fluorescence in the blue (PFO) and yellow (F8BT) regions. The spectra corresponding to F8BT are similar in both buffer and chloroform media and similar to those reported in the bibliography [35]. However, the emission spectrum of the PFO_CNPs was different from that obtained for the polymer in chloroform, with a clear red shift. This behavior has been described previously and is due to the fact that PFO can adopt two different phases—the disordered “glassy” phase and the more crystalline β-phase—which differ in the angles adopted between neighboring fluorene units. The “glassy” phase is adopted in good solvents, such as chloroform, and here these angles vary randomly. In contrast, in β-phase they adopt a fixed value of 180° corresponding to a well-defined planar chain conformation; this results in extended conjugation length, leading to red-shifted emission and improved optical performance [36]. The presence of β-phase in the PFO_CNPs can be confirmed from Figure 1 by the absorption peaks at 403 nm and 436 nm, as well as by the pronounced emission peaks at 440, 466 and 501 nm. Similar spectra have been obtained for PFO confined in different nanoparticles [37,38].

Time-resolved fluorescence spectroscopy was used to record the fluorescence decays of both PFO and F8BT CNPs in TRIS buffer. The fluorescence profiles were fitted to a three exponential decay model, and the mean fluorescence lifetime <τ> was calculated from this fit using Equation (1) (Figure S2). Values of 122 ps and 641 ps were calculated for PFO and P8BT_CNPs nanoparticles, respectively. The fact that the <τ> determined for the PFO_CNPs is longer than that reported for PFO in the “glassy” phase (97 ps), supports the suggestion that part of the polymer is adopting the β-phase inside the nanoparticle, which is characterized by a longer lifetime [37].

**Figure 2:**
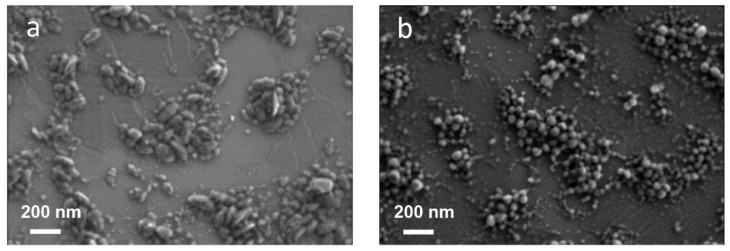
Representative images of scanning electron microscopy (SEM) of PFO_CNPs (**a**) and F8BT_CNPs (**b**).

The size, polydispersity and surface charge of the prepared CNPs were determined using DLS and zeta potential (ZP) measurements (Table 1). As for ZP, high negative values were obtained which guarantees appropriate suspension stability for both nanoparticles. Furthermore, the fact that these nanoparticles were negatively charged suggests that they could be incorporated into hydrogels through electrostatic interactions, given the cationic nature of the polymeric network. Moreover, the size and shape of the CNPs were determined by SEM (Figure 2). The results indicate that PFO_CNPs presented an elongated or ellipsoidal shape, in according with the β-phase presence in the nanoparticles [38]. In contrast, F8BT_CNPs were rather spherical and both had a size in agreement with those obtained in the DLS experiments.

To determine the most appropriate concentration of CNPs for the preparation of fluorescent hydrogels, increasing quantities of CNPs were added to a TRIS buffer solution, and emission spectra were collected after each addition. Figure S3 shows the rise in the fluorescence signal as the concentration of nanoparticles increased. As can be seen, the enhancement was initially linear and started to curve until reaching a maximum; it then began to decrease. This behavior was probably due to inner filter effects and mutual reabsorption between nanoparticles, leading to self-quenching. From these results, we selected 6 µM as the optimal concentration of CNPs to be incorporated into the hydrogels.

### 3.2. Preparation and Swelling Behavior of PFO@AETA and F8BT@AETA Hydrogels

Fluorescent hydrogels of PFO (PFO@AETA) and F8BT (F8BT@AETA) were prepared as described in Materials and Methods. As a first immobilization strategy, the ex situ method (Scheme 2a) was used. For this, a previously prepared hydrogel was dehydrated and swollen with a 6 µM suspension of CNPs until complete absorption, whereby it can be assumed that nearly 100% of the nanoparticles were entrapped. For the second strategy (in situ method in Scheme 2b), the CNPs suspension was mixed with AETA, MBA and photoinitiator before being irradiated under a UV lamp until the nanocomposite hydrogel was formed. On subsequent washing of the hydrogel, no fluorescence was detected in the wash water, confirming that the nanoparticles remain inside the hydrogel, probably through electrostatic interactions.

**Figure 3:**
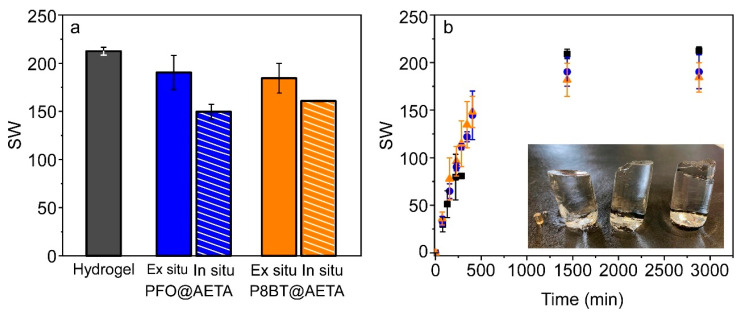
Maximum swelling degree of AETA hydrogel, PFO@AETA ex situ and situ, and F8BT@AETA ex situ and in situ (**a**). The corresponding swelling evolution of AETA hydrogel (black) and ex situ PFO@AETA (blue) and F8BT@AETA (orange) (**b**). Inset: Digital image of the dried hydrogel and the fully swollen hydrogel, ex situ PFO@AETA, and F8BT@AETA (from left to right). Error bars represent the standard deviation of three replicates.

The swelling degree of PFO@AETA and F8BT@AETA hydrogels prepared by both in situ and ex situ methods was explored and compared to that of the hydrogel in absence of CNPs. Experiments were performed at room temperature, as described in Materials and Methods, by placing the five dried hydrogels in an excess of Milli-Q water and allowing them to swell. As the water was absorbed by the hydrogels, their weight increased until the equilibrium swelling weight was reached (Figure 3). The results show that all five hydrogels greatly increased in volume, with respect to the dry hydrogel, demonstrating a high swelling capacity. The hydrogel that reached the highest degree of swelling, as determined from Equation (2), was the hydrogel in the absence of nanoparticles. In contrast, the nanocomposite hydrogels prepared by the in situ method, with either PFO or F8BT, showed the lowest degree of swelling, though swelling was also very noticeable. The swelling rate was also similar in the absence and presence of nanoparticles, as shown in Figure 3b for ex situ PFO@AETA and F8BT@AETA hydrogels. The inset in Figure 3b shows that the presence of CNPs inside the hydrogels hardly changed their final appearance. The results are in agreement with those obtained by Martin-Pacheco et al. [16] when they anchored negatively charged graphene QDs in a similar hydrogel and observed no significant changes in swelling behavior. These results show that CNPs hardly modify the structure of the polymeric network, at least using the ex situ method.

### 3.3. Fluorescence Properties of PFO@AETA and F8BT@AETA Hydrogels

**Figure 4:**
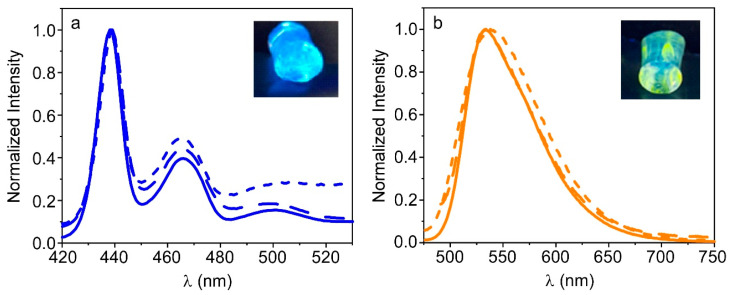
Normalized fluorescence emission spectra of PFO (**a**) and F8BT (**b**) CNPs in a solution of TRIS buffer (solid line), immobilized in hydrogels in situ (dotted line) and ex situ (dashed line). Insets: Digital image of PFO@AETA (**a**) and F8BT@AETA (**b**) hydrogels taken under irradiation with UV light (365 nm).

The above experiment allows characterization of the swelling properties of the hydrogels formed, but does not ensure that they exhibit fluorescent properties. To verify this, fluorescence spectra were recorded by placing cylindrical portions (~0.75 cm diameter) of each hydrogel in the fluorescence cuvette, and comparing them with those of the CNPs in buffer. All nanocomposite hydrogels exhibited a good fluorescence signal, confirming that nanoparticles were present inside them, though the signal intensity was lower than that observed in the buffer. Specifically, immobilized PFO_CNPs showed about four times less fluorescence intensity, while for F8BT_CNPs the reduction was one and a half times compared to solution. These differences could be attributed to the combination of different phenomena, such as inner filter effects, scattering from hydrogel, or self-quenching due to local concentration effects imposed by encapsulation. Figure 4 shows the normalized emission spectra of these hydrogels, as well as that of the CNPs in buffer, to clarify the differences between them. It can be seen that the hydrogels prepared by the ex situ method show spectra very similar to those of the nanoparticles in solution. In the case of PFO@AETA, the presence of β-phase is confirmed from the spectrum, evidencing that the PFO_CNPs are preserved inside the hydrogel, probably electrostatically interacting with the cationic polymeric network. A major difference was observed in the fluorescence spectra of the nanocomposite hydrogels prepared by the in situ method. The bands were slightly wider and, in the case of PFO, showed a tail towards longer wavelengths that could suggest aggregation of the nanoparticles. In addition, when observing the fluorescence of the hydrogels under the UV lamp (inset in Figure 4), we noticed that. in the case of F8BT@AETA. the emission was not homogeneous, as if the F8BT_CNPs were not equally distributed throughout the hydrogel. Furthermore, we observed that, after a few days, most of the fluorescence came from the bottom of the hydrogel, whether the hydrogel was prepared in situ or ex situ (Figure S4). This result suggests that either the nanoparticles do not interact effectively with the polymeric network, or the F8BT is released from the SDS, with the latter remaining bound to the network.

**Figure 5:**
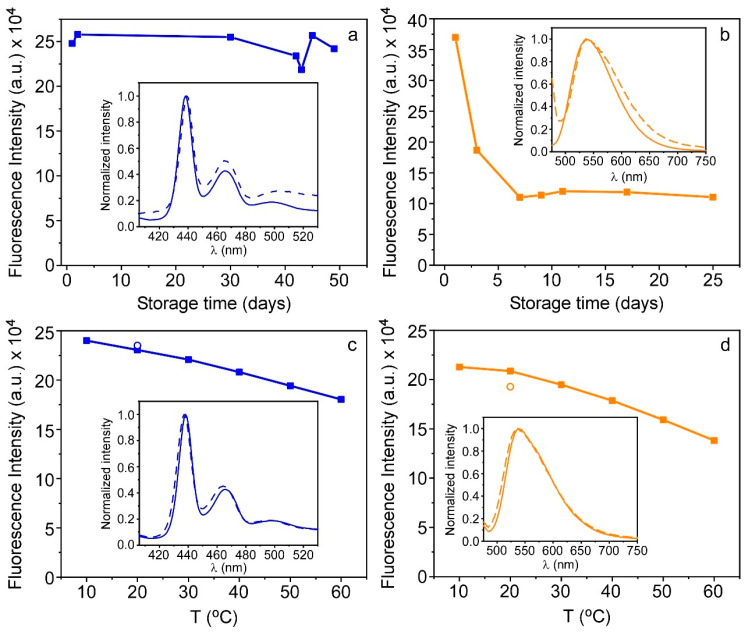
Effect of storage time and temperature on the stability of freshly prepared PFO@AETA (**a**,**c**) and F8BT@AETA (**b**,**d**) hydrogels measured at the fluorescence intensity maximum. The empty circles in (**c**,**d**) indicate the fluorescence intensity recovered after the heat treatment. Insets: Normalized fluorescence emission spectra at the first day (solid line) and the last day (dashed line) (**a**,**b**), and at 10 °C (solid line) and 60 °C (dashed line) (**c**,**d**).

In view of these results, we selected the ex situ method as the most suitable for the preparation of the fluorescent hydrogels; we then explored the fluorescence properties of PFO@AETA and F8BT@AETA hydrogels as a function of time, to better understand the above results. To this end, freshly prepared fluorescent hydrogels were stored at 4 °C and their emission spectra were recorded on different days. Figure 5a shows that the fluorescence intensity of PFO@AETA, measured at the emission maximum, remained stable for more than one month. However, the shape of the spectrum changed in a similar way to that observed for the in situ method, showing an increase in the second peak as well as a tail towards longer wavelengths; this could suggest a slight aggregation of the nanoparticles inside the hydrogel. Regarding F8BT@AETA, the fluorescence intensity strongly decreased during the first few days, broadening the spectrum; this supported the previous hypothesis that F8BT does not interact effectively with the polymeric network (Figure 5b). The temperature effect was also explored by recording the fluorescence spectra of freshly prepared hydrogels from 10 to 60 °C. For PFO@AETA, the decrease in fluorescence intensity observed with temperature increase was attributed to the increased probability of non-radiative transitions previously reported in polyfluorenes [27], since the initial fluorescence intensity was recovered after cooling (Figure 5c). In contrast, for F8BT@AETA, the effect of temperature was stronger and the initial intensity was not recovered, which supports the instability of F8BT_CNPs inside the hydrogel (Figure 5d).

Fluorescence decays were recorded in both PFO@AETA and F8BT@AETA hydrogels; however, due to the instability of the latter, it was not possible to obtain a good fit of the curves, so fluorescence lifetimes could only be determined for PFO@AETA hydrogels (Figure S2). Results show that the mean fluorescence lifetime <τ> for PFO entrapped in the hydrogel (PFO@AETA <τ> = 222 ns), is slightly higher than that calculated for the nanoparticles in solution. This increase in the mean lifetime within the hydrogel could be attributed to a higher presence of the β-phase [37], which could be correlated with the slight increase observed in the 466 nm fluorescent band (Figure 4a).

Taking into account these results, we concluded that F8BT@AETA hydrogels are not stable, at least under the preparation condition used in this work, so these materials were discarded for use in sensing applications. In contrast, PFO@AETA hydrogels could be good candidates for this kind of application, as long as their storage stability is improved.

### 3.4. Storage of PFO@AETA as Xerogels

**Figure 6:**
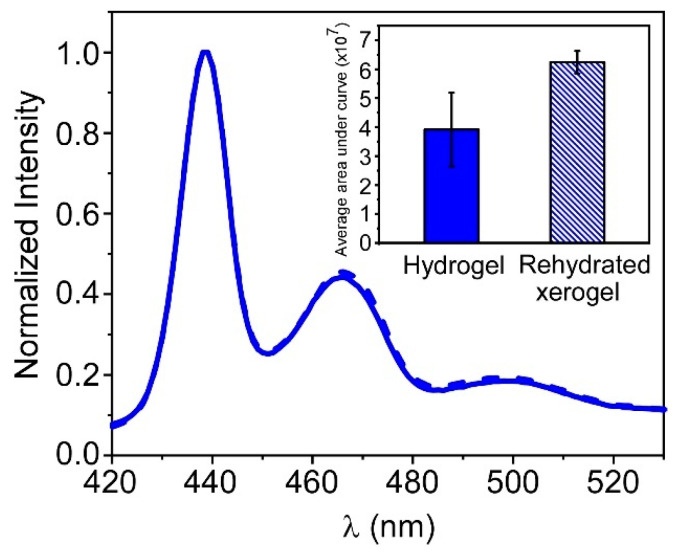
Normalized fluorescence emission spectra of PFO@AETA hydrogels (solid line) and rehydrated xerogels (dashed line). Inset: Average area under the (non-normalized) spectrum of different portions (n = 3) of hydrogels and rehydrated xerogels (𝜆_x_ = 396 nm). Error bars represent the standard deviation of the three replicates.

In recent work, we demonstrated the ability of AETA hydrogels to encapsulate enzymes and keep them stored in xerogel form, retaining their conformation and activity once rehydrated [28]. With this in mind, a series of PFO@AETA hydrogels were prepared and their fluorescence spectra recorded before and after being oven-dried and rehydrated to the initial volume (Figure 6). The shape and position of the spectrum were preserved after rehydration, and a higher signal and less variability in the fluorescence intensity were found (inset in Figure 6). The same behavior was recurrently observed in this study, suggesting that drying and subsequent swelling redistribute the nanoparticles more homogeneously; this maximizes their fluorescence emission, probably because they are more spread apart.

**Figure 7:**
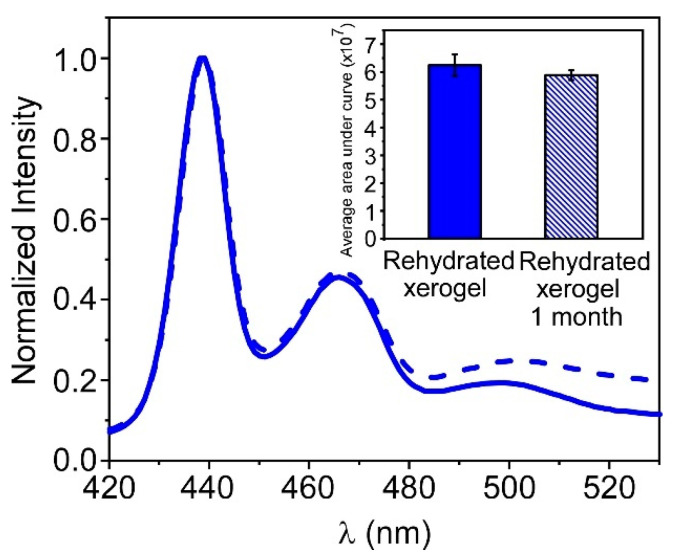
Normalized fluorescence emission spectra of rehydrated PFO@AETA xerogels stored at room temperature for one day (solid line) and one month (dashed line). Inset: average area under the (non-normalized) emission spectra of different portions (n = 3) of rehydrated xerogels stored for one day and one month (𝜆_x_ = 396 nm). Error bars represent the standard deviation of the three replicates.

The above result suggests that the material can be stored as a nanocomposite xerogel, improving its properties. To confirm this assumption, we tested if long-term storage as xerogel altered the fluorescence properties of PFO@AETA. For this purpose, portions of PFO@AETA xerogels were rehydrated after being stored at room temperature for thirty days; the fluorescence spectra were compared with hydrogels, stored as xerogels for only one day (Figure 7). The results show that both the shape of the spectrum, at least in the first 2 peaks, and the overall signal intensity were practically maintained. This indicates that xerogel is a good way to store fluorescent hydrogels at room temperature; moreover xerogel takes up little space, making fluorescent hydrogels easy to transport. The results suggest, therefore, that these materials could have potential applications in the field of detection.

### 3.5. PFO@AETA as a Sensing Platform

In previous works, researchers have exploited the ability of *p*-nitrophenol (PNP) to quench the fluorescence intensity of blue-emitter cationic polyfluorenes, to develop biosensors for phosphate detection or the screening of α-glucosidase inhibitors [29,39].

**Figure 8:**
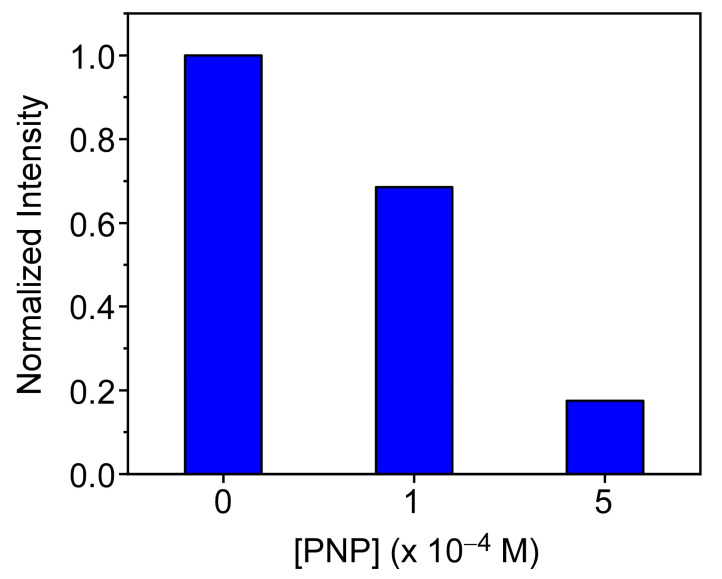
Effect of PNP on the fluorescence intensity of PFO@AETA hydrogels (λ_x_ = 396 nm, λ_m_ = 438 nm).

To study whether the developed material fluorescence is also quenched in the presence of PNP, PFO@AETA xerogels slices (~0.07 g) were incubated with solutions containing different PNP concentrations (0, 100 and 500 µM), until total absorption of the solution. The fluorescence spectra of the three hydrogels were then recorded, and their intensities at the emission maximum were compared. Figure 8 shows that PNP is able to largely quench the fluorescence of PFO@AETA, especially at concentrations of 500 µM, where more than 80% of the initial signal is lost. These results, therefore, suggest that the developed hydrogel can be used as a sensing platform in enzymatic reactions involving PNP as the final product.

**Figure 9:**
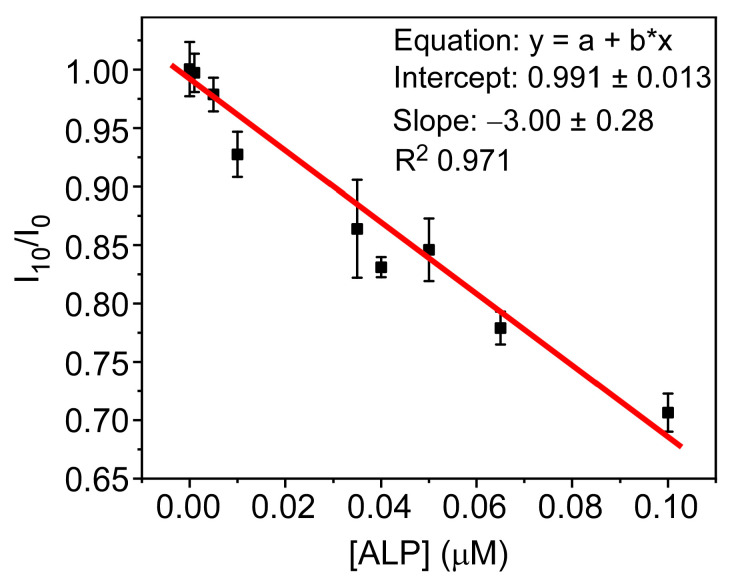
ALP calibration curve and fit parameters. Fluorescence quenching was measured 10 min after the addition of each ALP concentration (I_t = 10_/I_t = 0_) to partially rehydrated PNPP@PFO@AETA xerogels. Error bars represent the standard deviation of three replicates.

As a proof of concept, we investigated the possibility that PFO@AETA could be used for the detection of alkaline phosphatase (ALP), since ALP catalyzes the hydrolysis of *p*-nitrophenyl phosphate (PNPP) to PNP. For the practical application of the sensor, the incorporation of the substrate PNPP inside the PFO@AETA hydrogel is desirable. To this end, PFO@AETA xerogel was swollen with a solution of PNPP (0.5 mM) until complete absorption. Hydrogels were then oven-dried and stored at room temperature until their use. Afterwards, the suitability of the co-encapsulated system PNPP@PFO@AETAxerogels to detect ALP activity was evaluated. For this purpose, identical slices of the xerogel were partially rehydrated with 0.9 mL of buffer; 0.9 mL of ALP solutions containing different concentrations of the enzyme were then added, and the fluorescence intensity was measured as a function of time. The results, presented in Figure S5, show that, in the absence of the enzyme, the fluorescence signal experienced no significant variation over time. The fluorescence decrease only occurred in the presence of the enzyme, and its kinetics depends on the total ALP amount. This result suggests that hydrolysis of PNPP to PNP is taking place, and confirms the ability of the nanocomposite platform to detect the presence of ALP. To calibrate the response of the sensor to different ALP concentrations and determine its sensitivity the fluorescence quenching was collected after 10 min of incubation; it was then plotted as a function of the ALP concentration assayed (Figure 9). The plot was linear in the studied concentration range from 0 to 0.1 µM, with a limit of detection (LOD) of 21 nM, calculated from the following equation: LOD = 3σ/S, where S is the slope of the calibration curve and σ is the standard deviation of the blank.

In these preliminary assays, the developed device has demonstrated its ability to detect the presence of ALP in the nanomolar range in a similar way to other (disposable) ALP detection devices, some of which, although showing a slightly better detection limit, exhibit a lower linear response [40]. The achieved detection capacity of this first prototype, with a LOD equivalent to ALP ~40 IU/L, makes it suitable for the detection of abnormally elevated ALP levels in serum, which are associated with disease conditions [41]. It is interesting to note that, although sensors/assays with higher sensitivity to ALP than the one described in this work have been published in the literature (see, for example, a recent review by Shaban et al. [42]), the developed platform has not yet been optimized; this lack of optimization is due to the fact that our interest in this study was to verify that the synthesized nanocomposite material has the capacity to be used as a turn-off fluorescence sensor. We consider that, after minimal optimization, the device will be able to detect physiological levels of ALP in serum in an easy and low-cost way, as well as ALP activity in environmental samples, due to its interest as a bio-indicator and possible eutrophication biomarker [43,44]. Furthermore, we also want to highlight that the design of the device could allow not only the detection of this enzyme, but potentially also the detection of other enzymatic reactions involving PNP as an end product (such as those catalyzed by lipases, proteases or glucosidases). This could occur by simply modifying the substrate to the appropriate one for each enzyme.

## 4. Conclusions

In this study, we have developed and characterized two fluorescent acrylamide nanocomposite hydrogels containing conjugated polymer nanoparticles (CNPs) based on polyfluorenes—one emitting in the blue (PFO) and the other in the yellow (F8BT) regions—using a green protocol. In solution, both types of CNPs showed negative surface charge and diameters around 70 nm; for the PFO_CNPs, the presence of two phases (“glassy” phase and β-phase) was detected.

Two strategies for preparing the nanostructured hydrogels PFO@AETA and F8BT@AETA were explored: incorporation of CNPs during the polymerization process (in situ), and embedment after the hydrogel formation (ex situ). A slightly lower swelling degree was achieved for the in situ synthesized hydrogels, while the fluorescence spectra collected showed both a slight widening in the fluorescence bands of the CNPs and possible evidence of aggregation for PFO_CNPs. As a result, the ex situ method was selected.

Monitorization of the fluorescent properties of ex situ PFO@AETA and F8BT@AETA hydrogels showed that PFO@AETA presented higher stability and homogeneity, resulting in its selection as a sensing element. Moreover, drying of this hydrogel renders xerogels with improved fluorescent properties (higher signal and less variability), and allowed storage at room temperature for at least 30 days, which facilitates its use in the field of sensing.

Finally, as a proof of concept, PFO@AETA was used as a platform to specifically detect the enzyme alkaline phosphatase (ALP) in aqueous media, based on the ability of PNP (as a product of enzyme activity) to quench the fluorescence of the hydrogel. The developed prototype proved its ability to measure ALP in the nanomolar range with a LOD of 21 nM, showing great potential to quantify, after appropriate optimization, physiological ALP levels, as well as ALP activity in environmental samples. The main advantages of the synthesized material are its ease of handling, portability and great versatility. Its dimensions can be modified to adapt it to measurements in cuvettes, plate readers, etc. Sample analysis does not require the addition of substrate, as in other assays, as the substrate has been previously integrated into the platform. Furthermore, the design of the device would allow not only the detection of ALP, but potentially also the detection of other enzymatic reactions involving PNP as an end product, simply by modifying the substrate for the appropriate one for each enzyme.

## Figures and Tables

**Table 1:** Hydrodynamic diameter (d), polydispersity (PDI) and Zeta Potential (ZP) of PFO and F8BT CNPs.

Sample	d ± SD (nm)	PDI ± SD	ZP ± SD (mV)
PFO_CNPs	76.6 ± 0,5	0.203 ± 0.005	−45 ± 1
F8BT_CNPs	65.7 ± 0,9	0.155 ± 0.010	−62 ± 6

## Data Availability

Data reported in the study are available from the corresponding author on reasonable request.

## Supplementary Materials

The following supporting information can be downloaded at: https://www.mdpi.com/article/10.3390/bios13030408/s1, Figure S1: Normalized fluorescence emission of CNPs in chloroform; Figure S2: Fluorescence lifetimes of CNPs and PFO@AETA; Figure S3: Effect of increasing concentrations of CNPs; Figure S4: Digital image of F8BT@AETA. Figure S5: Fluorescence quenching kinetics of PFO@AETA with ALP.
