# Supplementary material for: Fluorescent Nanocomposite Hydrogels Based on Conjugated Polymer Nanoparticles as Platforms for Alkaline Phosphatase Detection
Source: Biosensors (Basel). 2023 Mar 21;13(3):408. doi: 10.3390/bios13030408 (PMC10046353; doi:10.3390/bios13030408)
Supplement: Supplementary file 1 [file biosensors-13-00408-s001.zip › biosensors-2259388-supplementary.pdf]

SUPPLEMENTARY MATERIAL

Fluorescent Nanocomposite Hydrogels Based on Conjugated Polymer Nanoparticles as Platforms for Alkaline Phosphatase Detection

Yolanda Alacid [1,2], Rocío Esquembre [1], Francisco Montilla [2], María José Martínez-Tomé [1,*] and C. Reyes Mateo [1,*]

[1] Instituto de Investigación, Desarrollo e Innovación en Biotecnología Sanitaria de Elche (IDiBE), Universidad Miguel Hernández, Avenida de la Universidad s/n, 03202, Elche, Alicante, Spain

[2] Departamento de Química Física and Instituto Universitario de Materiales de Alicante (IUMA), Universidad de Alicante, Carretera San Vicente s/n, 03690, Alicante, Spain

* Correspondence: mj.martinez@umh.es (M.J.M.T), rmateo@umh.es (C.R.M)

Keywords: Nanocomposite hydrogel; polyfluorene; fluorescent sensor; alkaline phosphatase; immobilization; portable device;

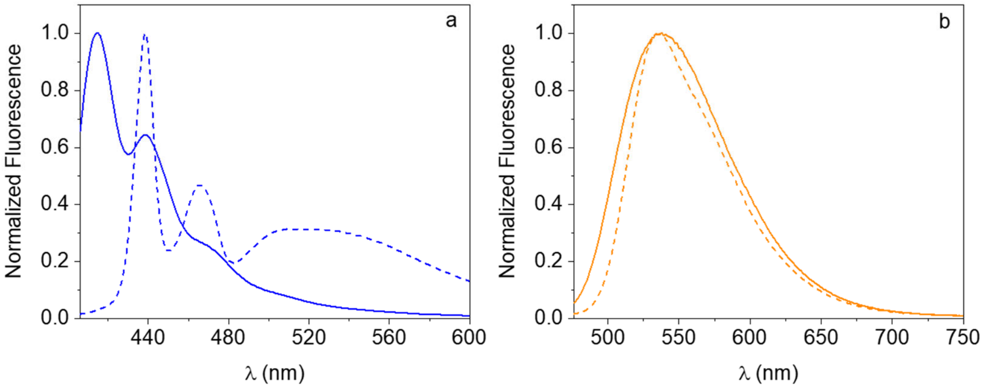

Figure S1. Normalized fluorescence emission spectra of PFO (**a**) and F8BT (**b**) NPs in chloroform (solid line) and TRIS buffer (dash line).

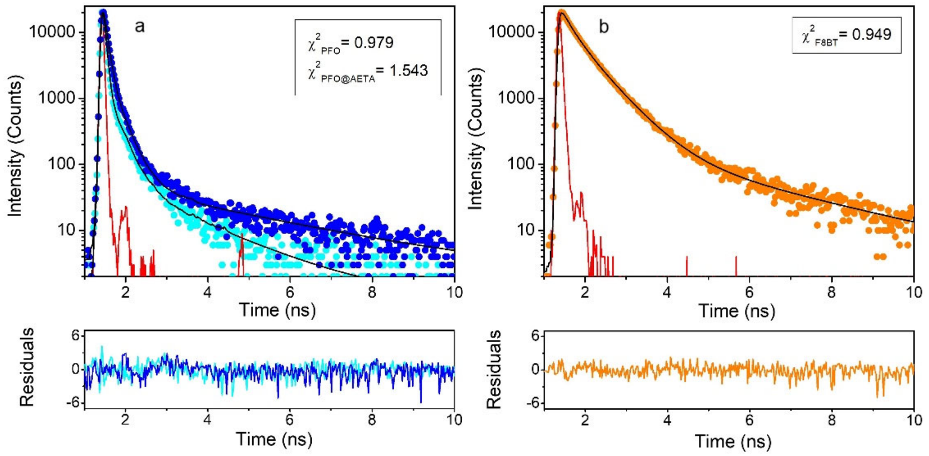

Figure S2. Semi-log plot of fluorescence decays recorded at 20°C for (**a**) FPO (cyan) and FPO@AETA (blue), and (**b**) F8BT (orange). The fitting curves are presented in black and the instrumental

response function measured with a LUDOX colloidal solution in red. The value of χ^2 and weighted residuals (lower panels), both used to asses the quality of the fits, are also shown.

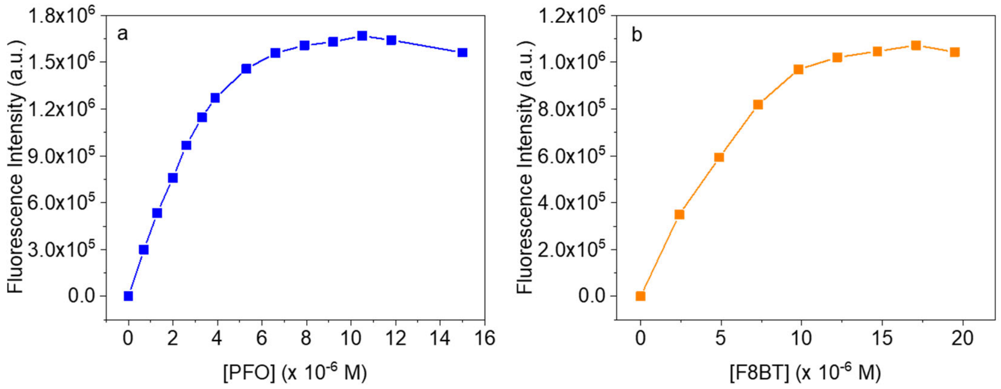

Figure S3. Influence of increasing concentrations of PFO_CNPs (**a**) and F8BT_CNPs (**b**) on the maximum fluorescence intensity to a buffered solution.

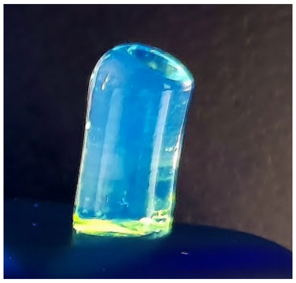

Figure S4. Digital image of ex situ F8BT@AETA hydrogel taken under irradiation with UV light (365 nm) after three days of preparation.

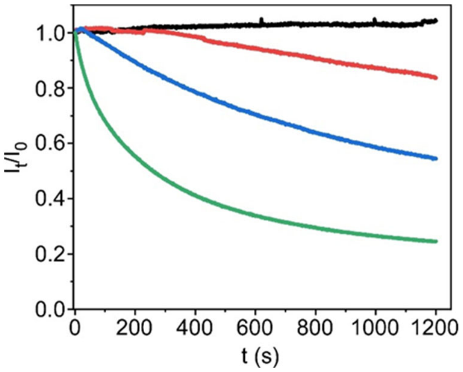

Figure S5. Fluorescence quenching kinetics (I_t/I_0) measured in PNPP@PFO@AETA hydrogels during their immersion in solutions containing concentrations of 0 (black), 0.01 (red), 0.1 (blue) and 3 (green) μM ALP.
